# Relationship between viral dose and outcome of infection in Atlantic salmon, *Salmo salar* L., post-smolts bath-challenged with salmonid alphavirus subtype 3

**DOI:** 10.1186/s13567-016-0385-2

**Published:** 2016-10-19

**Authors:** Jiraporn Jarungsriapisit, Lindsey J. Moore, Stig Mæhle, Cecilie Skår, Ann Cathrine Einen, Ingrid Uglenes Fiksdal, Hugh Craig Morton, Sigurd O. Stefansson, Geir Lasse Taranger, Sonal Patel

**Affiliations:** 1 Institute of Marine Research, Nordnesgaten 50, 5005 Bergen, Norway; 2 Department of Biology, University of Bergen, P. O. Box 7803, 5020 Bergen, Norway

## Abstract

Salmonid alphavirus subtype 3 (SAV3) causes pancreas disease (PD) and adversely affects salmonid aquaculture in Europe. A better understanding of disease transmission is currently needed in order to manage PD outbreaks. Here, we demonstrate the relationship between viral dose and the outcome of SAV3 infection in Atlantic salmon post-smolts using a bath challenge model. Fish were challenged at 12 °C with 3 different SAV3 doses; 139, 27 and 7 TCID_50_ L^−1^ of seawater. A dose of as little as 7 TCID_50_ L^−1^ of seawater was able to induce SAV3 infection in the challenged population with a substantial level of variation between replicate tanks and, therefore, likely represents a dose close to the minimum dose required to establish an infection in a population. These data also confirm the highly infectious nature of SAV through horizontal transmission. The outcome of SAV3 infection, evaluated by the prevalence of viraemic fish, SAV3-positive hearts, and the virus shedding rate, was positively correlated to the original SAV3 dose. A maximal shedding rate of 2.4 × 10^4^ TCID_50_ L^−1^ of seawater h^−1^ kg^−1^ was recorded 10 days post-exposure (dpe) from the highest dose group. The method reported here, for the quantification of infectious SAV3 in seawater, could be useful to monitor PD status or obtain data from SAV3 outbreaks at field locations. This information could be incorporated into pathogen dispersal models to improve risk assessment and to better understand how SAV3 spreads between farms during outbreaks. This information may also provide new insights into the control and mitigation of PD.

**Electronic supplementary material:**

The online version of this article (doi:10.1186/s13567-016-0385-2) contains supplementary material, which is available to authorized users.

## Introduction

Salmonid alphavirus (SAV), also referred to as Salmon pancreas disease virus (SPDV), is the aetiological agent of Pancreas disease (PD). PD adversely affects the production of salmonids in many countries in Europe and North America [1–5]. It is considered to be one of the most economically important diseases affecting salmonid production in Norway [6]. PD outbreaks in Norway occur during the seawater phase of both Atlantic salmon, *Salmo salar* L., and rainbow trout, *Oncorhynchus mykiss* (Walbaum), production cycles [7]. SAV belongs to the family *Togaviridae*, genus *Alphavirus*. The SAV genome is approximately 12 kilobases, and consists of a positive-sense single-stranded RNA encoding genes for structural (E1-E3 and capsid), 6K, and non-structural proteins (nsP1–nsP4) [8]. Six subtypes of SAV (designated SAV 1–6) have been identified based on partial sequences of the E2 and nsP3 genes [9], although this classification is not completely in agreement with serology-based criteria [10, 11]. Up until 2011, SAV3 had been the only subtype reported to cause PD in Norwegian aquaculture [7, 12]. Then, in 2011, the first case of SAV subtype 2 (SAV2) was detected [13], apparently following its introduction to Norway in 2010 [14, 15]. SAV2 has been reported to cause a milder form of PD than SAV3 [15–17], although serological reactivity between these two subtypes has been shown to be similar [10].

PD occurs mainly via horizontal transmission [14, 16, 18, 19], whereas vertical transmission has not been convincingly demonstrated [20, 21]. According to currently available scientific information, infected farmed salmonids are the main reservoir of SAV [14]. In Norway, restrictions on the movement of infected fish are in place in order to prevent the spread of PD. However, waterborne transmission of SAV via water currents cannot be prevented by movement restrictions. Viral shedding of SAV3 into seawater has been demonstrated from experimentally infected fish after intra-peritoneal injection [22] and bath immersion [23]. Mucus and faeces have also been suggested as routes of SAV shedding and transmission [16]. In addition, previous studies have shown that SAV1 can survive in non-sterile seawater for up to 14 days at 10 °C [24] and that only slight changes in SAV2 titre occur after incubation at 37 °C for 1 h [25]. These survival data, taken together with previous studies showing that there is a high risk of contracting PD from neighbouring farms during an active outbreak [18], strongly suggest that SAV transmission by water currents is possible and even likely. However, to date, there is a lack of quantitative information regarding viral shedding and the infectious dose required to induce infection in populations.

Experimental infection and monitoring of disease progression is one of the ways to obtain a deeper understanding of disease transmission [26, 27]. However, monitoring disease progression on the same individual is not always a practical approach for experimental animals where animal welfare must be considered. Hence, monitoring the stages of infection and pathogen transmission is often based on the results from different individuals. More knowledge regarding the relationship between viral dose and the outcome of the infection, viral shedding from fish, and survival of SAV in the environment is of utmost importance for the development of effective PD management strategies. This information can be incorporated into hydrodynamic modelling of SAV dispersal during outbreaks (including direction and distance) to assess the risk of PD spread, and may also contribute to the design and application of better control measures.

In the present study, the aim was to determine the relationship between the viral dose and the outcome of the SAV3 infection in Atlantic salmon post-smolts by using a previously established bath challenge model in seawater [23]. The bath challenge model allows the SAV infection to proceed by the natural route, it ensures the accuracy of the exposure time, and it allows for control of the viral dose. In this study, the effects of different doses on viral shedding, prevalence of infected fish, and viral load in the blood and in the heart were measured. These parameters were used to infer both the minimum infectious dose of virus that can induce infection in the population and the viral shedding rates. These are the key parameters that can be used in a viral dispersal model in order to improve the risk assessment of SAV transmission during PD outbreaks.

## Materials and methods

### Fish and rearing conditions

Atlantic salmon were supplied by SalmoBreed, Osterøy, Norway and transported in freshwater to the fish rearing facility at Industrial and Aquatic Laboratory (ILAB), Bergen High Technology Centre, Bergen, Norway, for production of post-smolts. The fish were screened by the supplier and tested negative for SAV, Infectious salmon anaemia virus (ISAV), Piscine orthoreovirus (PRV), Infectious pancreatic necrosis virus (IPNV) and Piscine myocarditis virus (PMCV). Experimental fish were unvaccinated Atlantic salmon post-smolts with an average weight of 50.6 ± 6.8 g and an average length of 16.3 ± 0.8 cm. Seawater (34‰) supplied throughout the experimental period was filtered through a 20 μm filter, UV-sterilized, and maintained at a constant temperature of 12 °C. The seawater flow rate and oxygen saturation were maintained at 300 L h^−1^ and >80%, respectively. Fish were fed to satiation and acclimatized in seawater for 9 days before the bath challenge. The shedder fish were bath anaesthetized with a mixture of metomidate (10 mg L^−1^) and benzocaine (60 mg L^−1^) before handling, and all fish were euthanized with a mixture of metomidate (10 mg L^−1^) and benzocaine (160 mg L^−1^) before tissue sampling.

### Experimental design and bath challenge

The bath challenge model with SAV3 in seawater was carried out as described previously [23], with some modifications. Briefly, 180 fish that served as shedder fish, were divided equally between three 150-L tanks containing seawater. Seven days prior to bath challenge, the shedder fish were intramuscularly injected with 10^3^ TCID_50_ of SAV3 per fish. On the day of the bath challenge, the water flow to the three shedder tanks was stopped for 1 h, after which the shedder fish were removed and euthanized. The seawater containing SAV3 (SAV3-seawater) from all shedder tanks was mixed to prepare three doses of SAV3 as follows: (i) undiluted SAV3-seawater (High dose) was transferred into Tanks 7 and 8; (ii) a 1:5 dilution of SAV3-seawater (Medium dose) was transferred into Tanks 4, 5 and 6; and (iii) a 1:75 dilution of SAV3-seawater (Low dose) was transferred into Tanks 1, 2 and 3. Then eighty-one unvaccinated fish were randomly allocated into each of the 8 identical 150-L seawater tanks described above. Each tank contained 120 L of the respective SAV3 doses (Low, Medium or High), and the fish were bathed for 6 h. The tanks were continually aerated, and the oxygen levels were closely monitored during challenge. After 6 h the water flow was resumed. All tanks were monitored for an hour after the water flow was restarted to ensure welfare of the fish. This study was approved by the Norwegian Animal Research Authority (NARA) and carried out in strict accordance with the guidelines.

### Salmonid alphavirus (SAV)

The SAV3 isolate used for infection of shedder fish in the present study originated from Atlantic salmon heart [28] and was kindly provided by Øystein Evensen, Norwegian University of Life Sciences, Faculty of Veterinary Medicine and Biosciences. SAV3 was propagated in Chum salmon heart-1 (CHH-1) cells cultured in L-15 medium (Life Technologies, UK) supplemented with 2% FBS (PAA, France) and Penicillin–Streptomycin-Amphotericin B (PSA) (Lonza, USA) at 15 °C. SAV3 was harvested 7 days after inoculation when a cytopathic effect (CPE) was observed. Quantification of virus stock was performed using end-point dilution assay on CHH-1 cells, and TCID_50_ mL^−1^ was calculated [29].

### Tissue sampling

Eight fish per tank were sampled at each sampling time-point. Weight and length were recorded. Blood was collected from the caudal vein into a microtube without anticoagulant at 7, 10, 14 and 21 dpe. Sera were collected by centrifugation (9500 *g*, 10 min) after storage of blood at 4 °C overnight. At 3, 7, 10, 14 and 21 dpe, hearts were dissected out of the fish and divided into 2 halves. One half was snap frozen in liquid nitrogen for RT-qPCR, while the other half was fixed in 10% neutral buffered formalin for histology. At 14 dpe, pancreas tissue associated with the pyloric caeca was also sampled and fixed in the same way as the hearts. Tissue samples for histology were also taken from fish in the Low dose tanks at 21 dpe, since we expected that this dose would result in a low prevalence of SAV3-positive individuals and/or a delay in detection of histopathological changes. A few samples were unfortunately lost during sample processing due to technical reasons.

### Water sampling

Duplicate samples (2 × 1 L) of seawater were collected from all three doses on the day of bath challenge. In addition, seawater was also collected from all 8 experimental tanks at 7, 10, 14, 17 and 22 dpe (1 × 1 L from each tank). Quantitation of the amount of SAV3 in the seawater samples was performed as described previously [22, 23] with some modifications. Briefly, water filtration based on the VIRADEL (virus-adsorption-elution) method was carried out using electropositive 1 MDS filters. One litre of seawater was collected, and concentrated by filtration. After filtration, the filters were placed upside down in petri-dishes containing 1.2 mL of L-15 (Life Technologies, UK) supplemented with 10% FBS (PAA, France) and agitated at 500 rpm for 15 min on an orbital shaker. The eluant was collected and passed through a 0.22 µm syringe filter unit (Merck Millipore, Germany). One hundred µL of the eluant was then transferred into a microtube containing 350 µL of lysis buffer (iPrep™ PureLink™ Total RNA Kit, Invitrogen, USA.) and stored at −80 °C prior to qPCR analysis. The remaining eluant was used in an end-point dilution assay on CHH-1 cells. The presence of SAV3 from eluant on the virus titration plate was confirmed by RT-qPCR [30] of the supernatant from cells showing CPE.

### RNA extraction

Total RNA was extracted from heart tissue with TRIzol^®^ reagent (Ambion) and an iPrep™ PureLink™ Total RNA Kit (Invitrogen, USA). Total RNA was quantified using a NanoDrop™-1000 spectrophotometer (Thermo Scientific). RNA was extracted from 100 µL of serum samples and filtered water eluants using lysis buffer and an iPrep™ PureLink™ Total RNA Kit according to the manufacturer’s instructions. The extracted RNA was in a final volume of 50 µL. Known amount of Nodavirus was added into serum as an internal control.

### In vitro synthesis of RNA standard

The viral RNA copy number was estimated using a SAV-specific RNA standard prepared as follows. SAV3 RNA was extracted as described in the previous section. A cDNA synthesis and PCR were performed to amplify a 576 bp fragment of the nsP1 gene using ThermoScript™ RT-PCR System and Platinum^®^ Taq DNA polymerase (Invitrogen, USA.) according to the manufacturer’s recommendation. The following primers were used: forward primer with a T7 RNA polymerase promoter (underlined): 5′ TAATACGACTCACTATAGGCTCACAGCTAACCCCTCCGCCGGCACTACAG 3′; and reverse primer: 5′ CGGCTCGAACCCGATCCAGTATACCACTCGCGTGCC 3′. The PCR fragment was cleaned with a QIAquick PCR Purification Kit (Qiagen). The molecular weight of the cleaned product was verified on a 1% agarose gel (Seakem LE Agarose, Lonza), the concentration measured using a NanoDrop™-1000 spectrophotometer, and the sequence verified. Purified PCR products were in vitro transcribed using T7MEGAscript Kit (Ambion) at 37 °C for 16 h followed by DNase treatment (TURBO DNA-free™ Kit, Ambion). Subsequently, the synthetic viral RNA (cRNA) was purified using RNeasy Mini Kit (Qiagen). The concentration of the purified cRNA was measured using a NanoDrop™-1000. The number of copies of the cRNA was calculated as described [31].

### cDNA synthesis and qPCR

The cDNA synthesis was performed using 200 ng total RNA from heart samples and 2 µL of cRNA (a ten-fold dilution series of cRNA to 10^1^–10^10^ copies) and RNA from serum and water samples in a 10 µL reaction volume (SuperScript VILO cDNA Synthesis Kit). A 1:10 dilution of cDNA was then used in a qPCR assay targeting the SAV nsP1 gene for detection of SAV3 [30]. The sample and standard mixture was prepared using TaqMan^®^ Fast Universal Master Mix (Applied Biosystems^®^) with 2 µL of diluted cDNA in a reaction mix containing 900 nM each of forward and reverse primers, and 250 nM of probe in a total volume of 10 µL on 384 well-plates. The qPCR assay was performed using ABI 7900HT Fast Real-Time PCR system (Applied Biosystems) and the temperature profile was adjusted as follows; activation at 95 °C for 20 s followed by 40 cycles of denaturation at 95 °C for 10 s, and annealing and extension at 60 °C for 20 s. Standards were included in each qPCR run and a threshold value of 0.1 applied to all samples. The standard curve and quantification of SAV3 RNA copies in samples were constructed and calculated automatically with the SDS software version 2.4.1 (Applied Biosystems, California, USA). The number of copies of SAV3 RNA in the sample was presented as per 2 µL of total RNA from serum, per 200 ng of total RNA from heart or per 2 µL of total RNA from concentrated seawater. Approximately 15% of randomly picked serum samples, representing all treatment groups and sampling points, were checked for nodavirus [32], which was added as an internal control, to validate the quality of the RNA extraction and cDNA synthesis and all the samples tested showed a satisfactory level of nodavirus.

### Histology

Pancreas and heart tissue from fish with SAV3-positive hearts, identified using qPCR, at 14 dpe (Low dose, *n* = 1; Medium dose, *n* = 5; High dose, *n* = 5) and at 21 dpe (Low dose, *n* = 1) were processed and embedded in paraffin wax. Three µm tissue sections were prepared, stained with Haematoxylin-Erythrosin-Saffron (HES) and examined by light microscopy.

### Data analysis

A Spearman’s rank-order correlation was used to determine the correlation between log_10_ viral loads in serum and heart after SAV3 infection from Medium and High dose using Statistica 12 (StatSoft, Inc., OK, USA). Data from the Low dose was not included as very few SAV3-positive samples were detected.

Fulton’s condition factor (K-factor) was calculated using the following formula, K-factor = 100 × weight (g)/[fork length (cm)]^3^, A t test was performed to analyze differences between K-factors using Statistica 12.

The estimated virus shedding rate in each experimental tank was calculated from virus titre, as measured by the end-point dilution assay, using the formula given below. This estimation is presented in units of TCID_50_ h^−1^ kg^−1^. The virus titre in the sample was back-calculated to the concentration in each of the experimental tanks using a concentration factor. The calculation was based on the following assumptions: virus was being constantly shed by fish in the population; virus particles were equally distributed in the seawater; the flow rate was constant; and the virus recovery from seawater samples was close to 100%. The virus titre in the sample was first calculated as described below. The flow rate and biomass in the tank were also taken into account in the final calculation.

$$\begin{aligned} {\text{Estimated shedding rate }}\left( {{\text{TCID}}_{50} {\text{h}}^{ - 1} {\text{kg}}^{ - 1} } \right) \hfill \\ = \frac{{{\text{TCID}}_{50} {\text{L}}^{ - 1} {\text{of seawater}} \times {\text {concentration factor }}\left( {\text{L}} \right) \times \frac{{{\text{flow rate }}\left( {{\text{L h}}^{ - 1} } \right)}}{{{\text{volume of seawater in tank }}\left( {\text{L}} \right)}}}}{{ {\text{remaining biomass in tank }}\left( {\text{kg}} \right)}}. \hfill \\ \end{aligned}$$

In this experiment, a concentration factor of 150 was used, and was calculated as a result of the volume of seawater in the experimental tank (150 000 mL) divided by the volume of the eluant recovered from the filter, which was approximately 1 mL (1000 times).

A Kruskal–Wallis test was used to compare prevalence of SAV3 nsP1-positive sera and hearts, also the log_10_ viral load in sera and in hearts between the three doses using Statistica 12.

## Results

### Bath challenge

Fish exposed to all three experimental doses of virus by bath challenge were successfully infected with SAV3. However, there was also a degree of variation between replicates depending on the viral dose. The SAV3 titre of the Low, Medium and High viral doses used for 6-h bath challenge, were 7, 27 and 139 TCID_50_ L^−1^ of seawater, respectively (Table 1). Some mortality was also observed in the Low (4 fish), Medium (4 fish) and High (1 fish) dose groups, but only three of these fish from Medium and High dose groups were subsequently shown to be SAV3-positive by qPCR.

**Table 1:** **Overview of dose–response from bath challenge of SAV3 in Atlantic salmon post-smolt**

Treatment	SAV3 exposure dose (TCID_50_ L^−1^ of seawater)	Prevalence of viraemic fish (%)	Prevalence of SAV3-positive heart (%)	Viral shedding rate in tank-seawater (TCID_50_ h^−1^ kg^−1^)
Low	7	4	0	1364
Medium	27	21	25	7113
High	139	81	63	24 129

Prevalence of viraemic fish, prevalence of SAV3-positive heart and viral shedding rate in the tank-seawater at 10 dpe are shown for each infection dose.

### Fulton’s condition factor (K-factor)

**Figure 1:**
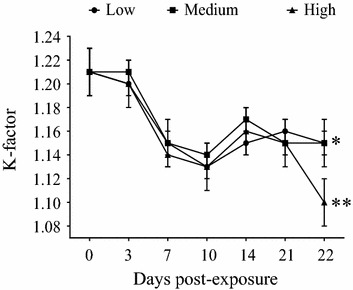
**Fulton’s condition factor (K-factor).** Mean ± SEM from Atlantic salmon post-smolts bath challenged with Low (black circle), Medium (black square) and High (black triangle) doses of SAV3. Comparison of K-factor between the beginning (0 dpe) and the end of the experiment (22 dpe) within the same dose group was carried out by using the t test and significant difference was observed. *p < 0.05, **p < 0.01.

The condition factor in the present study was between 0.90 and 1.46 and K-factors between the three doses at any sampling time were not statistically different. Overall, the K-factor decreased in all groups during the experiment, and this decrease was detected as early as 7 dpe in all three doses. Significant differences in K-factors were observed in all three doses, between the beginning (0 dpe) and the end of the experiment (22 dpe) (Figure 1).

### Relationship between viral load in sera and in hearts

The viral load in sera and hearts was tested to see if a correlation between these two parameters could be demonstrated. As there were very few SAV3-positive fish in the Low dose group they were excluded from this test. Spearman’s correlation coefficients demonstrated a strong positive correlation during the early phase of infection in the Medium (10 and 14 dpe) and the High (7, 10 and 14 dpe) dose groups (Table 2). A closer examination of the data at the level of individual fish showed that, generally, the increase in viral load was initially observed in serum and then in the heart. This was followed by a decrease in the viral load in serum and a slight increase or stabilization of the viral load in the heart.

**Table 2:** **Relationship between log**_**10**_ **viral loads in serum and heart from Medium and High dose**

Days post-exposure (dpe)	*r*_*s*_	
	Medium	High
7	na (24)	0.818 (16)**
10	0.765 (24)**	0.922 (16)**
14	0.826 (24)**	0.709 (16)**
21	0.116 (17)	0.431 (16)

Spearman’s correlation coefficient (*r*_*s*_) and number of sample in brackets.

na: not applicable.

** *p* < 0.01.

### Outcome of SAV3 infection

In this section, an overview of the results will be described first, followed by a more detailed description of the results from each viral dose.

The present study aimed to generate data on the relationship between viral dose and the outcome of SAV3 infection in Atlantic salmon post-smolts after a waterborne exposure to SAV3, in addition to studying the progress and severity of the infection. Results from 10 dpe showed that, as the viral dose was increased, the prevalence of viraemic fish, prevalence of SAV3-positive hearts, and viral shedding rate also increased (Table 1).

**Figure 2:**
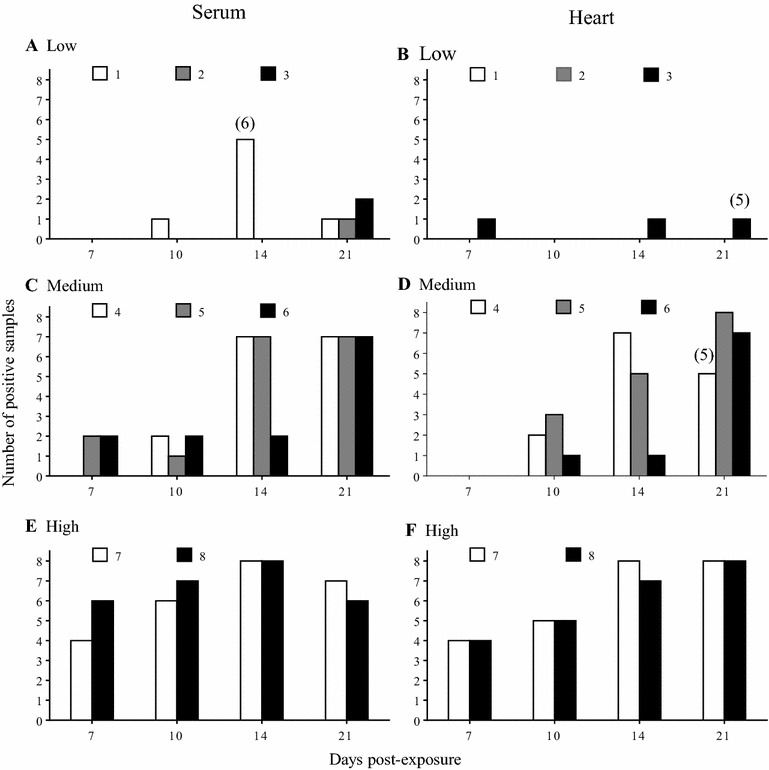
**Prevalence of infection following experimental bath challenge with SAV3 in post-smolts.** Prevalence of viraemia in sera (**A**,** C** and** E**), and SAV3-positive hearts (**B**,** D** and** F**) from the Low (**A** and** B**), Medium (**C** and** D**) and High (**E** and** F**) doses. Bars (white, grey and black) represent triplicate or duplicate tanks from the Low (1, 2 and 3) and Medium (4, 5 and 6) and High (7 and 8) doses. No hearts were positive for SAV3 at 3 dpe and are thus not shown. Numbers in brackets over columns in** A**,** B** and** D** indicate the number of samples analysed when it deviated from *n* = 8.

**Figure 3:**
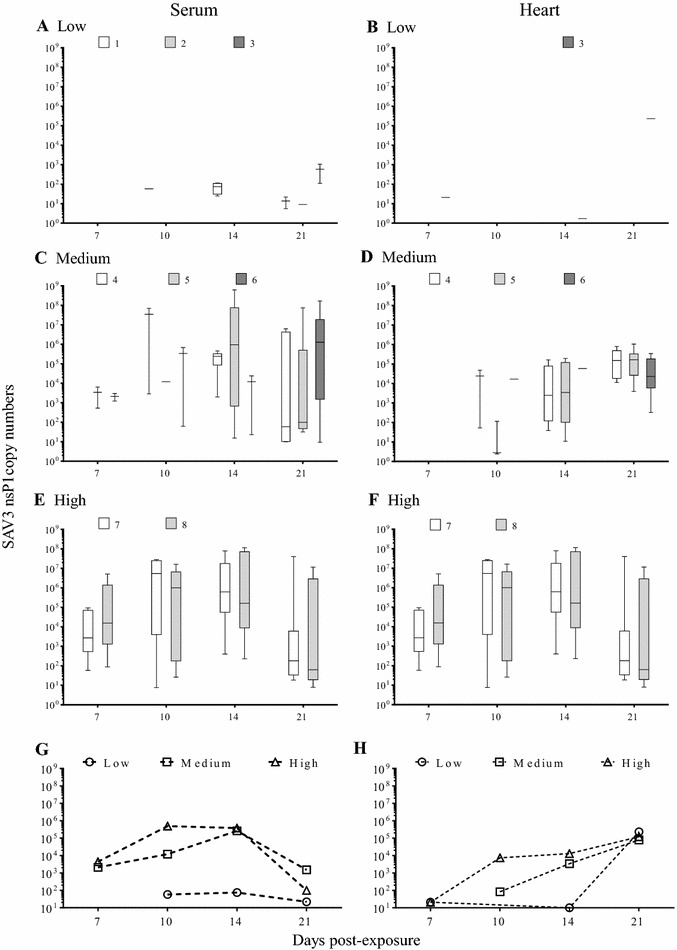
**Viral load measured by RT-qPCR following experimental bath challenge with SAV3 in post-smolt.** The results represent viral load in sera (**A**,** C** and** E**) and hearts (**B**,** D** and** F**) from Low (**A** and** B**), Medium (**C** and** D**) and High (**E** and** F**) doses. In each graph results are presented either as Box and whisker plots (median, quartiles and range) when 4 or more individuals per group were positive; whiskers without boxes when only 2 or 3 individuals per group were positive; a dash when a single positive individual per group was positive. Copy numbers are per 100 µL of serum or per 200 ng of total heart RNA. Line graphs with dashed lines represent the average medians of the viral load in sera (**G**) and heart (**H**) over the experimental period from all three doses.

The prevalence of viraemic fish and SAV3-positive hearts were positively correlated with viral dose, especially between 7–14 dpe (Figure 2). Viral load in sera was high in the period of 10–14 dpe in Medium and High dose then decreasing trends were observed at 21 dpe for all three doses (Figure 3G). Viral load in hearts from all three doses showed that the viral load in the positive fish reached a comparable level towards the end of the experiment, regardless of viral dose (Figure 3H).

Virus shedding could be detected using both RT-qPCR and end-point dilution assays. In addition, our data showed that end-point dilution assays detected virus in water samples more frequently than RT-qPCR (Additional file 1). Importantly, the end-point dilution method detects live virus in samples of epidemiological importance in the spread of the disease. The viral shedding data derived from the end-point dilution assays will be discussed in more detail below.

The severity of the histopathological lesions observed also appeared to correlate with SAV3 dose. The PD-associated histopathological lesions in the heart were most prominent in individuals exposed to the High dose. Overall, a viral load of ≥10^5^ SAV3 RNA copies per 200 ng of total RNA from heart corresponded to the observation of PD-related histopathological lesions in heart.

#### Low dose

*Prevalence*: The prevalence of viraemic fish and SAV3-positive hearts varied among the replicate tanks (Figures 2A and B; Table 3). SAV3 was detected at 10, 14 and 21 dpe in serum (Figure 2A) and at 7, 14 and 21 dpe in heart (Figure 2B). In addition, viraemic fish were detected only from Tank 1 at 10 and 14 dpe, while at 21 dpe fish in all 3 replicate tanks were viraemic (Figure 2A). In addition, SAV3-positive hearts were detected only from Tank 3, with a slight increase in prevalence over time (Figure 2B; Table 3).

**Table 3:** **The percentage prevalence of SAV nsP1 in sera and hearts as an average of 3 or 2 tanks per group and 95% confidence intervals (CI)**

Dose dpe	Sera						Hearts	
	7	10	14	21	7	10	14	21
Low								
Mean	0	4.2	27.8	16.7	4.2	0	4.2	6.7
95% CI	0–14.3	0.1–21.1	9.7–53.5	4.7–37.4	0.1–21.1	0–14.3	0.1–21.1	0.1–23.8
Medium								
Mean	16.7	20.8	66.7	87.5	0	25.0	54.2	95.8
95% CI	4.7–37.4	7.1–42.2	44.7–84.4	67.6–97.3	0–14.3	9.8–46.7	32.8–74.5	76.2–100
High								
Mean	62.5	81.2	100	81.2	50.0	62.5	93.8	100
95% CI	35.4–84.8	54.6–96.0	79.4–100	54.4–96.0	24.7–75.4	35.4–84.8	69.8–99.8	79.4–100

dpe: days post-exposure.

**Figure 4:**
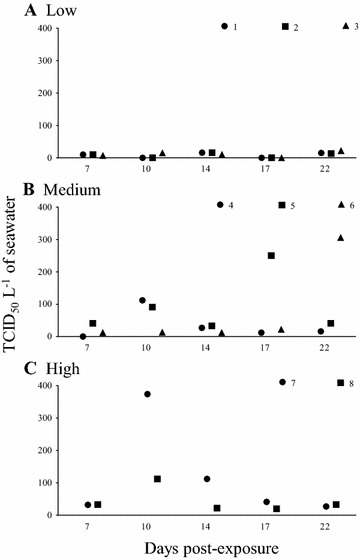
**Viral shedding of SAV3 from bath challenged post-smolts.** Each of the symbols (black circle, black square, black triangle) represent SAV3 titre (TCID_50_ L^−1^ of seawater) from 1L of seawater (one biological replicate), in the Low (1, 2 and 3), Medium (4, 5 and 6), and High (7 and 8) dose tanks. The same scale for the y-axes is shown in each plot to facilitate direct comparison between doses.

*Viral load*: SAV3 was not detected in sera at 7 dpe (Figure 3A), but it was detected in hearts from the fish in Tank 3 (Figure 3B). Low amounts of SAV3 (6–1.1 × 10^3^ copies) were detected in sera from fish at 10 (Tank 1), 14 (Tank 1) and 21 dpe (all replicate tanks) (Figures 3A and G). SAV3-positive hearts were detected at 7, 14 and 21 dpe with a substantial difference in viral loads (20, 2 and 2.3 × 10^5^ copies, respectively) (Figures 3B and H; Additional file 2). The average virus shedding was low or below the detection limit throughout the experimental period (0–22 TCID_50_ L^−1^ of seawater) (Figure 4A), resulting in a low shedding rate (0–2.90 × 10^3^ TCID_50_ h^−1^ kg^−1^) throughout the experimental period (22 days) with no shedding detected at 17 dpe (Table 4).

**Table 4:** **Estimated SAV3 shedding rate into tank-seawater from experimentally bath challenged Atlantic salmon post-smolts**

Dose (dpe)	Estimated shedding rate ± SEM (TCID_50_ h^−1^ kg^−1^ × 10^3^)				
	7	10	14	17	22
Low	0.76 ± 0.11 (0%)	1.36 (4%)	1.61 ± 0.28 (28%)	0 (na)	2.90 ± 0.36 (17%)
Medium	2.16 ± 1.20 (17%)	7.11 ± 3.02 (21%)	2.74 ± 0.74 (67%)	13.39 ± 10.96 (na)	21.25 ± 16.08 (88%)
High	2.66 ± 0.05 (63%)	24.13 ± 13.44 (81%)	7.73 ± 5.27 (100%)	4.74 ± 1.81 (na)	6.21 ± 0.02 (81%)

Mean percentage prevalence of viraemic fish is given in brackets.

na: not applicable; dpe: days post-exposure.

**Figure 5:**
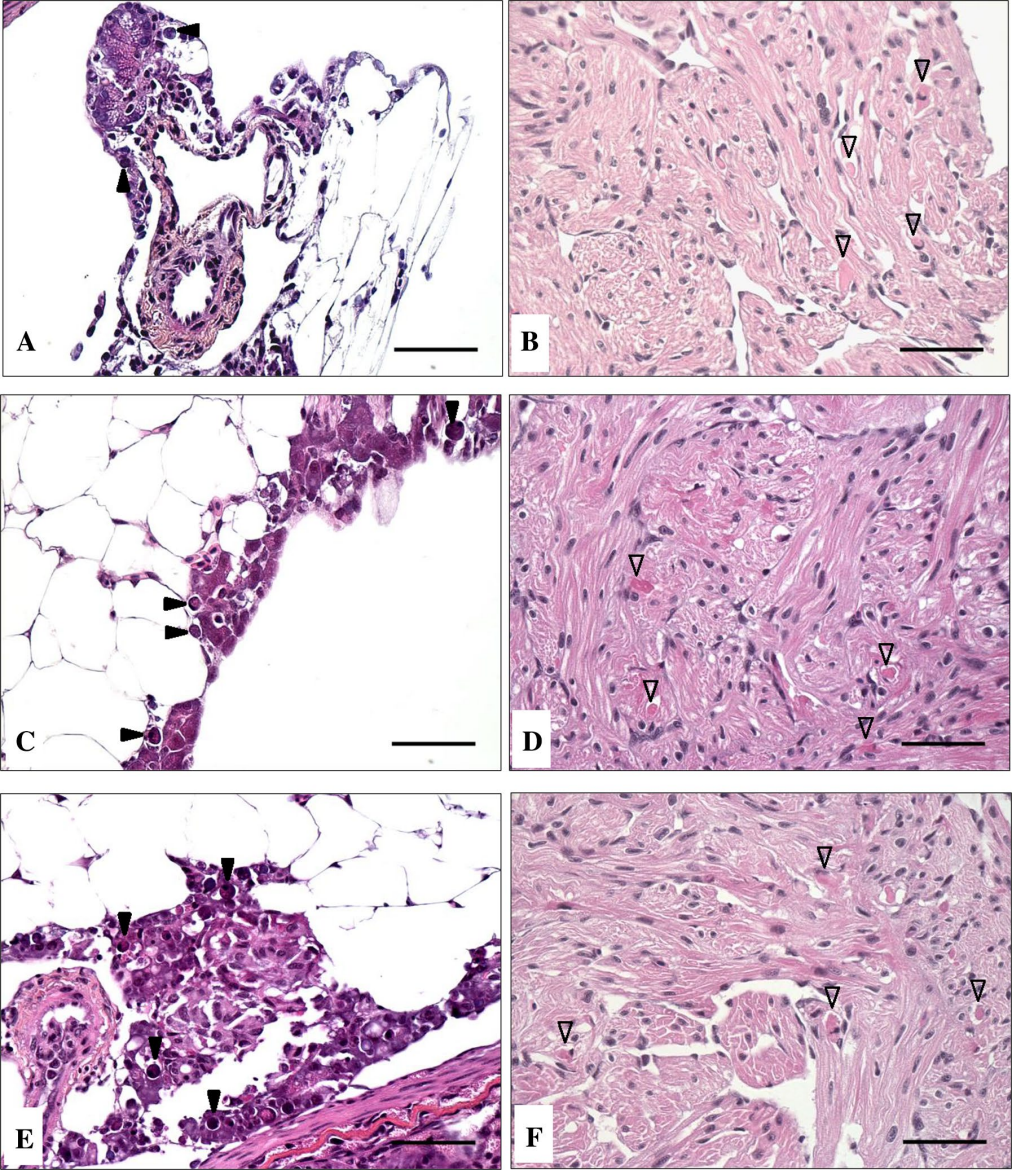
**Histopathological changes in the pancreas and heart of Atlantic salmon post-smolts following SAV3 bath challenge.** Necrosis of exocrine pancreatic cells (**A**,** C** and** E**) and degeneration of myocardial cells in the heart (**B**,** D** and** F**) in SAV3-positive fish from the Low dose (**A** and** B**) at 21 dpe, and from the Medium dose (**C** and** D**) and High dose (**E** and** F**) at 14 dpe. Solid arrowheads represent necrosis of pancreatic exocrine cells and open arrowheads represent myocardial degeneration. Scale bars represent 50 µm.

*Histopathology*: SAV3-positive fish from the Low dose did not show any of the characteristic PD-associated histopathological lesions in the pancreas or the heart at 14 dpe, PD-associated lesions were observed in the SAV3-positive fish from this group at 21 dpe (Figures 5A and B).

#### Medium dose

*Prevalence*: The prevalence of viraemic fish and SAV3-positive hearts varied among the three replicate tanks with less variation than for the Low dose group (Figures 2C and D). In general, fish in all three replicate tanks were SAV3-positive at all time-points. However, at 7 dpe SAV3 was detected in sera from fish in 2 out of 3 tanks, but the hearts were not yet positive (Figures 2C and D). The average prevalence of SAV3-positive sera and hearts increased over time reaching 88 and 96%, respectively, at 21 dpe (Table 3).

*Viral load*: SAV3 was detected in sera from fish in Tanks 5 and 6 at 7 dpe (Figure 3C) which coincided with detection of SAV3 in the tank-water (Figure 4B). However, SAV3 was not detected in hearts at 7 dpe (Figure 3D). SAV3 was detected in sera, hearts and water in all 3 replicate tanks from 10 dpe and onwards (Figures 3C, D; 4B). The viral load in sera increased between 7–14 dpe (2.1 × 10^3^, 1.2 × 10^4^ and 2.6 × 10^5^ copies at 7, 10 and 14 dpe, respectively), and then declined at 21 dpe (1.5 × 10^3^ copies) (Figure 3C; Additional file 2). The viral load in hearts increased throughout the experimental period (8.3 × 10^1^, 3.4 × 10^3^ and 7.9 × 10^4^ copies at 10, 14 and 21 dpe, respectively) (Figure 3D; Additional file 2). At 7 dpe the level of SAV3 in the water was low (26 TCID_50_ L^−1^ of seawater). The level of SAV3 was higher at 10 dpe (72 TCID_50_ L^−1^ of seawater) but then declined again at 14 dpe (24 TCID_50_ L^−1^ of seawater) (Figure 4B). In addition, there was an increase in average virus shedding after 14 dpe (95 and 121 TCID_50_ L^−1^ of seawater at 17 and 22 dpe, respectively) (Figure 4B, dashed line). In the Medium dose fish, the shedding rate was low but was higher than the shedding rate from the Low dose fish (Table 4).

*Histopathology* The PD-associated histopathological lesions in pancreas and heart tissue of SAV3-positive fish from the Medium dose group were observed at 14 dpe (Figures 5C and D). It is worth noting that a proportion of the samples showed more severe histopathological lesions in pancreas than in heart.

#### High dose

*Prevalence* The prevalence of viraemic fish and SAV3-positive hearts was relatively similar between the two replicate tanks (Figures 2E and F). In both replicate tanks SAV3 was detected in sera and hearts at all sampling time-points (Figures 2E and F). The average prevalence of SAV3-positive sera increased from 63% (7 dpe) to 81% (10 dpe), and peaked at 100% (14 dpe) before declined to 81% (21 dpe) (Table 3) while the average prevalence of SAV3-positive hearts increased over time (50, 63 and 94% at 7, 10 and 14 dpi, respectively) and reached 100% at 21 dpe (Table 3). The prevalence of viraemic fish and SAV3-positive hearts of High dose were significantly different from Low and Medium dose at 10 dpe (*p* < 0.05).

*Viral load* SAV3 was detected in sera, hearts and water in both replicate tanks from 7 dpe (Figures 3E, F; 4C). The viral load in sera increased from 4.4 × 10^3^ (7 dpe) to 4.9 × 10^5^ copies (10 dpe), then declined slightly to 3.8 × 10^5^ (14 dpe) and to 1.0 × 10^2^ copies (21 dpe) (Figure 3E; Additional file 2), whereas the viral load in hearts increased over time (21, 7.4 × 10^3^, 1.3 × 10^4^ and 1.2 × 10^5^ copies at 7, 10, 14 and 21 dpi, respectively) (Figure 3F; Additional file 2). The log_10_ sera from High dose was significantly different from Low and Medium dose at 7 and 10 dpe whereas it was significantly different from Low dose at 14 and 21 dpi (*p* < 0.05).

SAV3 was detected in water from the High dose group throughout the entire experimental period (Figure 4C). The pattern of virus shedding clearly showed only one peak at 10 dpe (243 TCID_50_ mL^−1^) in contrast to the shedding in the Medium dose tanks (Figures 4B and C). In the High dose, the shedding rate was comparable to the Medium dose at 7 dpe (2.66 × 10^3^ TCID_50_ h^−1^ kg^−1^) but a substantial increase was seen at 10 dpe (2.41 × 10^4^ TCID_50_ h^−1^ kg^−1^). This was followed by a decrease at 14 dpe (7.73 × 10^3^ TCID_50_ h^−1^ kg^−1^) and a low and stable rate of shedding was detected at 17 and 22 dpe (4.74 and 6.21 × 10^3^ TCID_50_ h^−1^ kg^−1^) (Table 4).

*Histopathology* SAV3-positive fish from the High dose group showed necrosis of exocrine pancreatic cells and degeneration of myocardial cells in the heart at 14 dpe (Figures 5E and F). The SAV3-positive fish from this group also showed the most severe PD-related histopathological lesions amongst the three doses.

## Discussion

In the present work, we utilised a recently established bath challenge model [23] to study the relationship between viral dose and the outcome of SAV3 infection in Atlantic salmon post-smolts. Based on the detection of SAV3 in sera, hearts, and tank-water samples as well as observation of typical PD-related histopathological changes in the pancreas and heart, the bath challenge model has proven to be a valuable model to study the natural infection route of SAV3. The decrease in Fulton’s condition factor reported here has also been observed in other SAV3 infection studies [15, 23, 33]. Mortality in experimental SAV infection is difficult to reproduce [16, 22, 23, 34], and only one cohabitation challenge study has demonstrated PD-related mortality (of approximately 15%) [15]. In the present study, mortality undisputedly caused by SAV3 infection was not observed, however, SAV3 infection may have been a contributing factor to the observed mortality.

The lowest concentration of SAV3 used in the 6 h-bath challenge (7 TCID_50_ L^−1^ of seawater) was able to cause infection in the challenged population as shown by detection of SAV3 in blood, heart, and in tank-water. Substantial variability between the replicate tanks was observed at this low SAV3 concentration, therefore, 7 TCID_50_ L^−1^ of seawater is possibly close to the minimum dose required for waterborne SAV3 infection of post-smolts of this size. In addition, from the three doses used in the bath challenge, we estimate the 50% infectious dose to be in the range of 27–139 TCID_50_ L^−1^ of seawater. This information may be useful in other dose–response studies.

In the present study, all the challenged fish were exposed to SAV3 at the same time, therefore, the infection was expected to develop homogeneously, especially during the early phase of disease progression. The failure to detect SAV3 in the hearts in the Low and Medium dose groups at early time-points might simply be explained by the SAV3 levels being below the level of detection of our assay. Alternatively, the prevalence of fish with SAV3-positive hearts could have been low. The detection of prevalence might be limited during the early phase of the experiment, which might explain the variability in detection especially in the low dose tanks. As the sampling continued, a higher percentage of the population sampled combined with the progress in disease development, may have increased the probability for prevalence detection. Therefore, improving the sensitivity of the detection method or increasing the number of samples analysed during surveillance programmes would likely improve the success of virus detection especially when the prevalence of SAV3-infected fish in the population is low.

The prevalence of viraemia in sera, prevalence of SAV3-positive hearts, and the virus shedding rates were used to determine the outcome of SAV3 infection. The degree of severity of the SAV3 infection in the challenged groups directly corresponded to the SAV3 dose in the bath challenge. Basically, a higher dose resulted in a higher percentage of infected fish in the population allowing the virus to spread throughout the entire population faster. In addition, the severity of PD-associated histopathological lesions also showed a similar correlation to the SAV3 exposure dose. The histopathological lesions in the heart were especially prominent in individuals with a SAV3 RNA copy number above 10^5^ per 200 ng of total RNA. This copy number value is similar to a previous study [28] (just above 10^6^ viral RNA copies per 1 µg of total RNA) although the method was somewhat different. Interestingly, a viral load above 10^5^ copies per 200 ng of total RNA in the heart might be an indication of the presence of PD-related lesions in the hearts of fish with PD. It has been reported that the level of SAV1 in infected hearts could be as high as 10^6^ copies at 11 days after intraperitoneal injection [35].

Viral shedding rates of fish populations vary depending on the initial virus dose and the stage of the infection. In this present study, the maximal rate of viral shedding of 2.4 × 10^4^ TCID_50_ h^−1^ kg^−1^ was observed in the High dose at 10 dpe. During PD outbreaks on fish farms, the biomass (kg) and the water current passing through a sea cage (L h^−1^) can be measured or estimated from the records. In addition, the prevalence of viraemic fish within a population and the virus shedding rate should also be possible to calculate. In the present study, the effect of viral dose on the prevalence of infected fish and the viral shedding rate at the population level was observed. When the prevalence of viraemic fish was taken into consideration for the calculation of virus shedding rates, the maximal shedding rates from the Medium and High dose at 10 dpe were similar (3–4 × 10^4^ TCID_50_ h^−1^ kg^−1^). Hence, we speculate that maximal virus shedding from individual fish is possibly constant or comparable in fish with similar characteristics (such as age, size, physiological condition, and genetic background). More research is clearly needed, but if our speculation is proved to be true, we may be able to better predict the virus shedding rate at the population level during natural outbreaks in real-time. A strong positive-correlation between viral loads in sera and hearts was observed in the High dose group during the first 14 dpe, indicating that higher viral loads in serum positively correlate with high viral loads in the heart early in the infection. A lower level of correlation at later time-points may indicate clearance of the virus from the bloodstream as specific antibody production can be observed 2–3 weeks after exposure to SAV [16, 34]. In contrast, the viral load in the hearts was relatively stable or slightly increased due to persistence of SAV in this organ [22, 23, 30]. At the end of the study, the Spearman correlation coefficient was markedly lower in the Medium dose group than in the High dose group. This suggests that clearance of the virus from the blood is more efficient when fish are exposed to a lower dose of virus. However, various stages of disease progression in this population may also contribute to the observed change in the correlation coefficient. This information would be valuable if it could be used to describe the overall status of PD at a population level.

Contagious individuals likely shed virus through mucus and faeces [16, 36] and infect the remaining individuals within the same population resulting in multiple sequential infections within the population. In the present study, the increase in viral shedding at 10 and 22 dpe in the Medium dose group illustrates this scenario. The increase in the prevalence of viraemic fish (from 67 to 88%) and SAV-3 positive hearts (from 54 to 96%) at later time-points supports the scenario of multiple rounds of infection within the population. This is undoubtedly the scenario during SAV outbreaks at aquaculture sites [37]. Thus, the disease status of individuals or populations resulting from exposure to low levels of virus requires information from multiple samples such as blood, pancreas, heart, muscle and water and may employ various techniques such as RT-qPCR, end-point dilution assay, water filtration, serology and histology.

In the present study, a method allowing SAV3 to be isolated from seawater samples [22, 23] was combined with a conventional end-point dilution assay to quantify the amount of infectious virus. Although it is difficult to demonstrate the efficiency of virus recovery by this method, recovery of virus was in the same log concentration in end-point dilution assay during optimization of the method (unpublished observations). Interestingly, quantification of virus using RT-qPCR was less sensitive than end-point dilution assay when SAV3 concentration in the water samples was low. Overall, water samples with viral titres below 100 TCID_50_ L^−1^ of seawater were not detected by RT-qPCR. However, the presence of SAV3 in the supernatants from the end-point dilution assay could be confirmed using RT-qPCR. The small amounts of virus in the starting material and the probability of selecting a non-representative fraction during sample processing, such as extraction of nucleic acid and cDNA synthesis, prior to the RT-qPCR assay, might contribute to the loss of sensitivity. Similarly, virus multiplication in cell culture and replication of samples in the end-point dilution assay could contribute to the increased sensitivity of this method. Nevertheless, the high sensitivity of RT-qPCR methods for SAV detection in tissue samples are well-known [34, 38] and this was also true in the present study as long as the virus concentration in samples was above the detection limit. In addition, RT-qPCR is a rapid detection method whereas the end-point dilution assay for SAV requires at least 3 days [39], but it also reflects the presence of infectious particles.

The method for quantification of infectious SAV3 in seawater samples reported here could be applied to field studies, although the presence of other viruses in the environment and the likely low amounts of SAV in seawater cages may complicate interpretation of the data and require further optimization of the method. Nevertheless, a preliminary attempt to apply this method for samples collected during a reported PD outbreak in the field confirmed that the method is applicable (unpublished observation). It would be very interesting to add the viral shedding data and the SAV3 survival data into a pathogen dispersal model in order to improve the risk assessment of PD transmission during a natural outbreak. Improving the accuracy of both disease transmission and risk assessment models may lead to better control and mitigation of PD. This is of utmost importance for the sustainable growth of the fish farming-industry.

## Additional files

**Additional file 1.** **Viral shedding into the tank-water from Atlantic salmon post-smolt following bath challenge with SAV3**. Low (●), Medium (■) and High (▲) doses measured by end-point dilution assay (solid lines) and qPCR (dashed lines). The unit of RNA copy number is per 2 µL of total RNA from concentrated water.

**Additional file 2.** **Viral loads in serum and heart.** The results are presented as medians and quartiles of the SAV3 nsP1 copy numbers per 200 ng total heart RNA or per 100 µL serum.

## Abbreviations

cRNA: synthetic RNA
dpe: days post-exposure
PD: pancreas disease
RT-qPCR: quantitative reverse transcription polymerase chain reaction
TCID_50_: 50% end-point tissue culture infectious dose
