# Supplementary figures and images for: Relationship between viral dose and outcome of infection in Atlantic salmon, Salmo salar L., post-smolts bath-challenged with salmonid alphavirus subtype 3
Source: Vet Res. 2016 Oct 19;47:102. doi: 10.1186/s13567-016-0385-2 (PMC5069985; doi:10.1186/s13567-016-0385-2)

## Slide 1
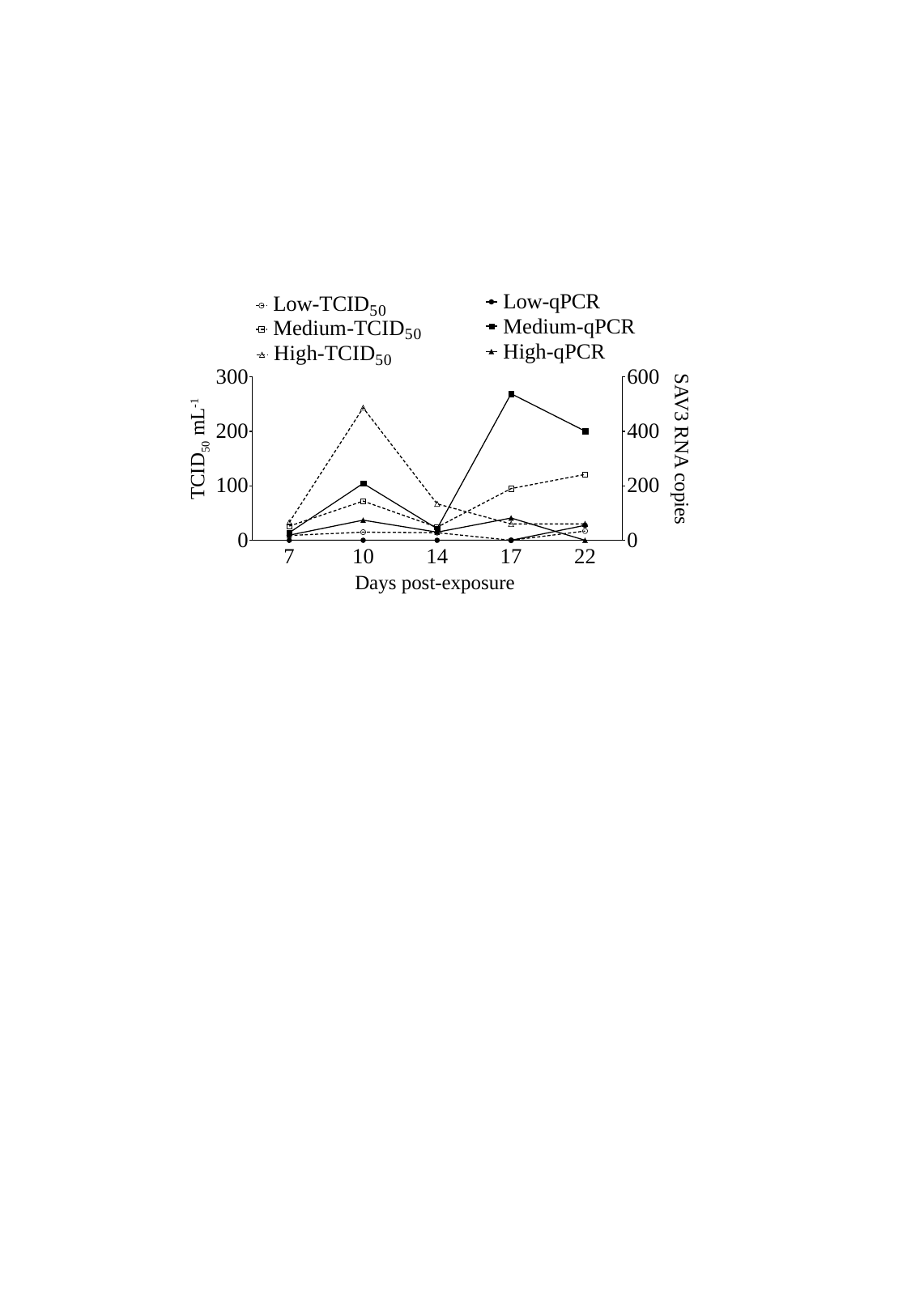

TCID50 mL-1
SAV3 RNA copies
Days post-exposure

Supplement: Supplementary file 1 — Additional file 1. Viral shedding into the tank-water from Atlantic salmon post-smolt following bath challenge with SAV3. Low (●), Medium (■) and High (▲) doses measured by end-point dilution assay (solid lines) and qPCR (dashed lines). The unit of RNA copy number is per 2 µL of total RNA from concentrated water. [file 13567_2016_385_MOESM1_ESM.pptx]
