# Supplementary material for: Relationship between viral dose and outcome of infection in Atlantic salmon, Salmo salar L., post-smolts bath-challenged with salmonid alphavirus subtype 3
Source: Vet Res. 2016 Oct 19;47:102. doi: 10.1186/s13567-016-0385-2 (PMC5069985; doi:10.1186/s13567-016-0385-2)
Supplement: Supplementary file 2 — Additional file 2. Viral loads in serum and heart. The results are presented as medians and quartiles of the SAV3 nsP1 copy numbers per 200 ng total heart RNA or per 100 µL serum. [file 13567_2016_385_MOESM2_ESM.pptx]

## Slide 1
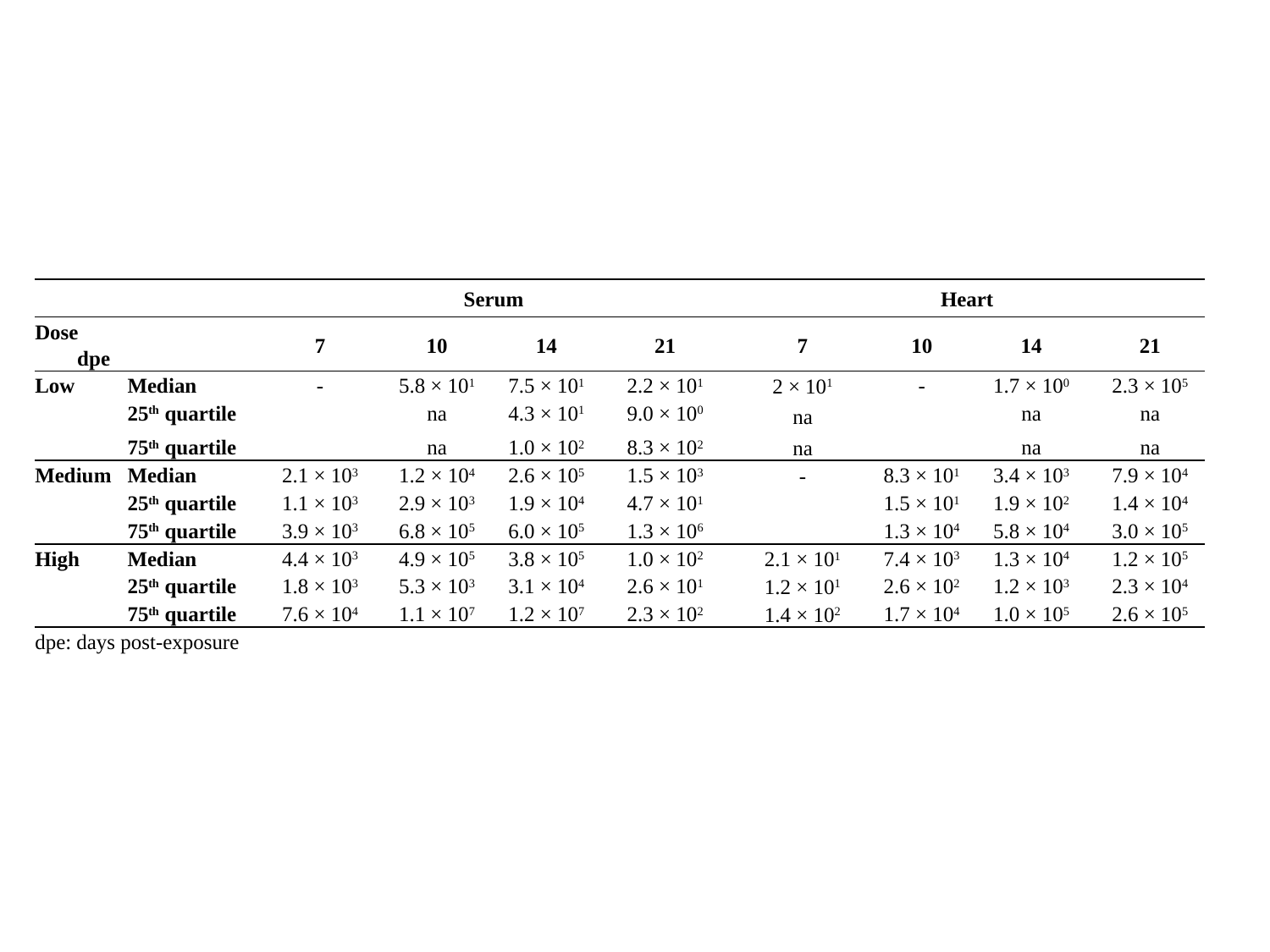

| | | Serum | | | | Heart | | | |
| --- | --- | --- | --- | --- | --- | --- | --- | --- | --- |
| Dose dpe | | 7 | 10 | 14 | 21 | 7 | 10 | 14 | 21 |
| Low | Median | - | 5.8 × 101 | 7.5 × 101 | 2.2 × 101 | 2 × 101 | - | 1.7 × 100 | 2.3 × 10^5^ |
| | 25th quartile | | na | 4.3 × 101 | 9.0 × 100 | na | | na | na |
| | 75th quartile | | na | 1.0 × 10^2^ | 8.3 × 10^2^ | na | | na | na |
| Medium | Median | 2.1 × 10^3^ | 1.2 × 10^4^ | 2.6 × 10^5^ | 1.5 × 10^3^ | - | 8.3 × 101 | 3.4 × 10^3^ | 7.9 × 10^4^ |
| | 25th quartile | 1.1 × 10^3^ | 2.9 × 10^3^ | 1.9 × 10^4^ | 4.7 × 101 | | 1.5 × 101 | 1.9 × 10^2^ | 1.4 × 10^4^ |
| | 75th quartile | 3.9 × 10^3^ | 6.8 × 10^5^ | 6.0 × 10^5^ | 1.3 × 10^6^ | | 1.3 × 10^4^ | 5.8 × 10^4^ | 3.0 × 10^5^ |
| High | Median | 4.4 × 10^3^ | 4.9 × 10^5^ | 3.8 × 10^5^ | 1.0 × 10^2^ | 2.1 × 101 | 7.4 × 10^3^ | 1.3 × 10^4^ | 1.2 × 10^5^ |
| | 25th quartile | 1.8 × 10^3^ | 5.3 × 10^3^ | 3.1 × 10^4^ | 2.6 × 101 | 1.2 × 101 | 2.6 × 10^2^ | 1.2 × 10^3^ | 2.3 × 10^4^ |
| | 75th quartile | 7.6 × 10^4^ | 1.1 × 10^7^ | 1.2 × 10^7^ | 2.3 × 10^2^ | 1.4 × 10^2^ | 1.7 × 10^4^ | 1.0 × 10^5^ | 2.6 × 10^5^ |
| dpe: days post-exposure | | | | | | | | | |
| | | | | | | | | | |
